# Fall risk-increasing drugs and falls: a cross-sectional study among elderly patients in primary care

**DOI:** 10.1186/1471-2318-14-40

**Published:** 2014-03-27

**Authors:** Veronica Milos, Åsa Bondesson, Martina Magnusson, Ulf Jakobsson, Tommy Westerlund, Patrik Midlöv

**Affiliations:** 1 Center for Primary Health Care Research, Institution of Clinical Sciences, Lund University, Lund, Sweden; 2 Department of Medicines Management and Informatics, Region Skåne, Sweden; 3 Clinical Pharmacology, Department of Laboratory Medicine, Lund University, Lund, Sweden; 4 University of Gothenburg, Gothenburg, Sweden; 5 Medical Products Agency, Department of Usage Uppsala, and Sahlgrenska Academy, Institute of Medicine, Department of Public Health and Community Medicine, Unit of Social Medicine, University of Gothenburg, Gothenburg, Sweden; 6 Department of General Medicine, Center for Primary Health Care Research, Institution of Clinical Sciences, Lund University, Malmö Clinical Research Centre (CRC), building 28, floor 11 Jan Waldenströms gata 35, Skåne University Hospital, 205 02 Malmö, Sweden

**Keywords:** Elderly, Falls, Prevention, Drug therapy, Fall risk-increasing drugs

## Abstract

**Background:**

Falls are the most common cause of injuries and hospital admissions in the elderly. The Swedish National Board of Health and Welfare has created a list of drugs considered to increase the fall risk (FRIDs) and drugs that might cause/worsen orthostatism (ODs). This cross-sectional study was aimed to assess FRIDs and their correlation with falls in a sample of 369 community-dwelling and nursing home patients aged ≥75 years and who were using a multi-dose drug dispensing system.

**Methods:**

Data were collected from the patients’ electronic medication lists. Retrospective data on reported falls during the previous three months and severe falls during the previous 12 months were collected. Primary outcome measures were incidence of falls as well as numbers of FRIDs and ODs in fallers and non-fallers.

**Results:**

The studied sample had a high incidence of both reported falls (29%) and severe falls (17%). Patients were dispensed a mean of 2.2 (SD 1.5) FRIDs and 2.0 (SD 1.6) ODs. Fallers used on average more FRIDs. Severe falls were more common in nursing homes patients. More women than men experienced severe falls. There were positive associations between number of FRIDs and the total number of drugs (p < 0.01), severe falls (p < 0.01) and female sex (p = 0.03). There were also associations between number of ODs and both total number of drugs (p < 0.01) and being community dwelling (p = 0.02). No association was found between number of ODs and severe falls. Antidepressants and anxiolytics were the most frequently dispensed FRIDs.

**Conclusions:**

Fallers had a higher number of FRIDs. Numbers of FRIDs and ODs were correlated with the total number of drugs dispensed. Interventions to reduce falls in the elderly by focusing on reducing the total number of drugs and withdrawal of psychotropic medications might improve the quality and safety of drug treatment in primary care.

## Background

Drug prescribing in patients aged ≥75 years increased by nearly 70% in Sweden between 1990 and 2010 [1]. A comprehensive Swedish register-based study showed that a high number of drugs in elderly patients is related to a higher risk of prescribing potentially inappropriate medications, as well as higher risks of side-effects and drug-drug interactions [2]. A meta-analysis of prospective studies indicated that almost 17% of hospital admissions in the USA were caused by adverse drug reactions [3]. Meanwhile, both Swedish and international studies have shown that a majority of hospital admissions related to inappropriate drug use could potentially be prevented [4].

Falls are the most common cause of injuries among patients older than 65 years. Seventy-three percent of hospital admissions of patients older than 65 years are due to falls [5]. Upper extremity fractures and hip fractures are the most common fall-related injuries that lead to emergency department visits [6]. A Swedish study showed that treatment with fall risk-increasing drugs (FRIDs) was very common (93%) among older hip fracture patients both before and after the fracture [7].

Today, there is a consensus definition of falls [8]. Several risk assessment tools are available to assess a hospitalised [9,10] or community-dwelling [11,12] patient’s risk of falling. The tools assess different clinical characteristics as confusion, dizziness, cognitive impairment or administered drugs. Although the causes of falls are multi-factorial, medications are an important risk factor that it might be possible to influence. The most common FRIDs are different types of psychotropic drugs, such as sedatives, hypnotics, antidepressants and antipsychotic medications, which can cause sedation, impaired balance and coordination. The use of selective serotonin reuptake inhibitors (SSRIs) has been associated with falls regardless of the presence of depressive symptoms [13]. Due to age-related physiological changes in blood pressure-regulating systems and cardiovascular co-morbidity, cardiovascular drugs may cause or worsen orthostatic hypotension and falls [1,14,15]. Anti-Parkinson’s disease and dopaminergic drugs might also increase the fall risk by causing or worsening orthostatic hypotension, dyskinesia or hallucinations [16]. Anticholinergic drugs, such as antihistamines and urological spasmolytics, may affect elderly patients’ cognitive skills and cause blurred vision, thereby increasing the fall risk [16].

There is clear evidence that polypharmacy and the use of psychotropic drugs, especially when combined with cardiovascular medications or present as therapeutic duplications, increase the fall risk [16-19]. Medications for night-time sedation, such as lorazepam and zopiclone, have been found to be the most frequently prescribed medications before a fall in general medicine inpatient units in Canada [20].

A meta-analysis of interventions aiming to prevent falls in the elderly showed that slow withdrawal of psychotropics significantly reduced the risk of falling and that prescribing modification programs for primary care physicians significantly reduced risk of falling [21].

The National Board of Health and Welfare (NBHW) in Sweden has produced a FRID list, and also a list of drugs causing or worsening orthostatic blood pressure, which is relevant for assessing the fall risk (Table 1) [1].

**Table 1:** Fall risk-increasing drugs (FRIDs) and drugs that may cause or worsen orthostatism (ODs) according to the list from the Swedish National Board of Health and Welfare (NBHW)

ATC* code	**Drugs/group of drugs**
**Increase the fall risk**	
NO2A	Opioids
N05A (NO5AN excluded)	Antipsychotics (lithium excluded)
N05B	Anxiolytics
N05C	Hypnotics and sedatives
N06A	Antidepressants
**May cause or worsen orthostatism**	
C01D	Vasodilators used in cardiac diseases
C02	Antihypertensives
C03	Diuretics
C07	Beta blocking agents
C08	Calcium channel blockers
C09	Renin-angiotensin system inhibitors
G04CA	Alpha-adrenoreceptor antagonists
N04B	Dopaminergic agents
N05A (NO5AN excluded)	Antipsychotics (lithium excluded)
N06A	Antidepressants

*Anatomical Therapeutic Chemical classification system.

According to the Swedish Central Bureau of Statistics, the proportion of the population 75 years or older was 9% in Sweden in 2012. Community-dwelling older adults and nursing home residents in Sweden use on average 8–10 different drugs [1]. A large proportion of them use the multi-dose drug dispensing (MDD) system. This system involves machine-packaging all the medications that the patient should take at any particular time together in small labelled plastic bags. This packaging is done at a regional pharmacy dispensing centre and means that nurses are not involved in drug dosage preparation [22]. The use of the MDD system ensures a more reliable source of a patient’s active medication list [23].

This study aimed to explore the association between the drugs on the NBWH list of FRIDs and ODs and falls in Swedish elderly community-dwelling and nursing home patients.

## Methods

### Patients and settings

Patients included in the study were users of the MDD system [23], aged 75 years and older, living in nursing homes or in their own homes with municipally provided home care.

Patient data were collected from a separate randomised controlled trial (RCT) examining whether multi-professional drug reviews including a pharmacist could improve the quality of pharmacotherapy among elderly primary health care patients [24]. At baseline, nurses completed a symptom checklist using the Pharmacotherapeutical Symptom Evaluation 20 (PHASE-20) tool [25] and sent the results to a pharmacist participating in the study. PHASE-20 includes 20 questions and is designed to identify drug-related symptoms (Additional file 1) [25]. For this study, all intervention and control patients from the aforementioned study [24] were included. Information on baseline characteristics, such as age, sex, residency, locomotion and blood pressure, was extracted from the PHASE-20 responses.

The study received ethical approval from the Regional Ethical Review Board in Lund (no. 2011/245).

### Data collection

The patients were recruited to the RCT between September 1 and December 16 2011.

Data collection for the present study was conducted between September 1 2012 and February 15 2013. Baseline drug lists were screened for FRIDs and ODs according to the NBHW list. To facilitate the identification of FRIDs and ODs, a list of all generic names and product names was created. All identified drugs were classified according to the Anatomical Therapeutic Chemical (ATC) classification system [26]. Every drug was counted as one with its unique ATC code regardless of the dosage or number of pills for the individual patient. Data on FRIDs and ODs were collected and analysed separately due to the distinction made by the NBHW and the fact that drugs from certain ATC groups (e.g. antipsychotics) appear on both the FRID and OD lists.

The data for reported falls and severe falls were collected. Reported falls were defined as falls during the past three months reported by the nurse in the patient’s PHASE-20 checklist evaluation. Severe falls were defined as falls leading to emergency visits at hospitals or hospital admission as a consequence of syncope, contusion or bone fracture during the previous year as documented in the patient’s EMR. Data on hospital admissions and hospital emergency visits relating to falls during the year prior to inclusion in the study were collected from the patient’s hospital electronic medical records (EMRs).

### Data analysis

Primary outcome measures were incidence of falls as well as numbers of FRIDs and ODs in fallers and non-fallers. The secondary outcome measure was distribution of drug types among FRIDs and ODs. Data were analysed using Student’s t-test and Fischer’s exact test for two-group comparisons, and multiple linear regression (backward method) analyses. In the two regression analyses FRIDs and ODs were used as the respective dependent variables while age, gender, place of living, number of drugs and severe falls were entered as independent variables. A significance level of a = 0.05 was chosen. All data were analysed using IBM SPSS version 20.0.

## Results

Seventy-six percent of the 369 included patients were women and the mean age was 87.4 (SD 5.7) years. A majority (76%) were living in nursing homes. Table 2 shows the baseline data for the patients.

**Table 2:** Baseline characteristics of the study sample

**Characteristic**	**Patient sample**
Patients, N	369
Female, N (%)	280 (76)
Age (years), mean (SD)	87.4 (5.7)
Residency, N (%)	
Nursing home	279 (76)
Community	90 (24)
Mean no. of drugs, N (SD)	11.8 (4.5)
No. of continuous drugs	9.5 (3.9)
No. of drugs as needed	2.3 (1.8)
Locomotion, N (%)	
Ambulatory	204 (72)
Chair-bound	76 (27)
Bed-bound	2 (1)
Blood pressure (mmHg), mean (SD)	
Systolic	130 (19.5)
Diastolic	70 (11.5)

The patients were prescribed a mean of 2.2 (SD 1.5) FRIDs according to the FRID list of the NBHW and 2.0 (SD 1.6) drugs from the OD list of the NBHW. Only 13% of the study sample had no drugs prescribed from the FRID or OD lists. Data collected from the PHASE-20 symptom checklist were available for all 369 patients. Almost four in ten patients experienced moderate to severe dizziness, unsteadiness or fatigue. Data about reported falls from the PHASE-20 assessment were only available for 275 patients (75%). Twenty-nine percent of these patients reported at least one fall in the three months prior to the PHASE-20 evaluation. More men reported falls during the past three months. There were no differences between patients who reported falls and those who did not fall during the past three months with regard to age, total number of drugs and place of living (Table 3).

**Table 3:** Comparisons between fallers and non-fallers regarding age, sex, place of living, number of drugs, FRIDs and ODs

**Outcome variable**	**Falls during the last 3 months before the symptom evaluation**			**Falls leading to emergency visits or hospital admissions during the last 12 months**		
	**Falls**	**No falls**	**P value**	**Falls**	**No falls**	**P value**
**Sex, N (%)**						
Male	31 (44)	39 (56)	<0.01^*^	4 (4)	85 (96)	<0.01^*^
Female	50 (24)	155 (76)		58 (21)	222 (79)	
**Age, mean (SD)**	87.2 (5.7)	87.2 (5.4)	0.97^**^	87.8 (5.6)	87.3 (5.7)	0.53^**^
**Place of living, N (%)**						
Nursing home	53 (26)	149 (74)	0.07^*^	56 (20)	223 (80)	<0.01^*^
Community	28 (38)	45 (62)		6 (7)	84 (93)	
**No. of drugs, mean (SD)**						
Total	11.5 (3.8)	11.8 (4.8)	0.58^**^	12.6 (4.4)	11.6 (4.5)	0.12^**^
Continuous use	9.5 (3.6)	9.2 (4.0)	0.64^**^	9.8 (3.5)	9.4 (3.9)	0.39^**^
As needed	2.0 (1.4)	2.5 (2.0)	0.01^**^	2.7 (2.1)	2.2 (1.6)	0.08^**^
**No. of FRIDs**^**1**^**, mean (SD)**						
Total	2.4 (1.5)	2.0 (1.4)	0.06^**^	2.7 (0.7)	2.0 (0.6)	<0.01^**^
Continuous use	2.0 (1.4)	1.6 (1.2)	0.02^**^	2.1 (1.4)	1.6 (1.3)	<0.01^**^
As needed	0.4 (0.6)	0.5 (0.7)	0.41^**^	0.5 (0.7)	0.4 (0.6)	0.13^**^
**No. of ODs**^**2**^**, mean (SD)**						
Total	1.8 (1.4)	2.0 (1.6)	0.26^**^	1.7 (1.5)	2.0 (1.5)	0.15^**^
Continuous use	1.6 (1.3)	1.7 (1.4)	0.38^**^	1.4 (1.2)	1.7 (1.3)	0.05^**^
As needed	0.2 (0.4)	0.2 (0.4)	0.28^**^	0.2 (0.4)	0.2 (0.4)	0.36^**^

*Fishers exact test.

**Student’s t-test.

^1^FRIDs = Fall risk-increasing drugs according to the NBHW.

^2^ODs **=** Drugs that may cause or worsen orthostatism according to the NBHW.

There were no significant differences between total number of drugs, number of FRIDs, number of ODs or blood pressure between community-dwelling and nursing home patients when performing a Student’s t-test.

Data for severe falls collected from the patients’ EMRs were available for all 369 patients. Seventeen percent had at least one severe fall during the previous year. Severe falls were more common in nursing home patients as compared to community-dwelling elderly patients. More women experienced severe falls.

Two multiple linear analyses with number of FRIDs and ODs as dependent variables were performed (Table 4). They showed positive associations between the number of FRIDs and the total number of prescribed drugs (p < 0.01) and severe falls (p < 0.01). Being female was associated with a higher number of FRIDs (p = 0.03). Associations were found between the number of ODs and both the total number of prescribed drugs (p < 0.01) and community dwelling (p = 0.02). No association was found between the number of ODs and the occurrence of severe falls.

**Table 4:** Regression models with FRIDs and ODs as dependent variables

**Dependent variable: FRIDs**					
**Model**	**Unstandardised coefficients**		**Standardised coefficients**	**Sig.**	**Model summary**
	**B**	**Std. error**	**Beta**		
Female sex	-0.340	0.162	-0.099	0.037	Adjusted R squared = 0.225
No. of drugs	0.145	0.015	0.442	<0.001	
Severe falls	0.515	0.187	0.130	0.006	
**Dependent variable: ODs**					
No. of drugs	0.191	0.015	0.542	<0.001	Adjusted R squared = 0.313
Severe falls	-0.432	0.186	-0.102	0.152	
Community living	0.392	0.162	0.106	0.016	

Seventy-four different drugs were prescribed to patients among the total number of 1533 FRIDs. The five most frequently prescribed drugs among the FRIDs and ODs in the NBHW lists had the ATC codes N (Nervous System) (54.1%) and C (Cardiovascular System) (45.6%). The frequency and percentage of the different ATC groups among prescribed FRIDs are presented in Table 5. For the FRID list of the NBHW, the five most frequent prescribed FRIDs were oxazepam (n = 151), citalopram (n = 113), zopiclone (n = 104), mirtazapine (n = 68) and zolpidem (n = 44).

**Table 5:** Frequency and percentage of ATC groups for FRIDs and ODs according to NBHW lists

**FRIDs**		
ATC*code	**Frequency**	**%**
N06A (Antidepressants)	238	29.4
N05B (Anxiolytics)	194	24.0
N05C (Hypnotics and sedatives)	187	23.1
N02A (Opioids)	142	17.5
N05A (Antipsychotics)	47	5.8
Total	808	100
**ODs**		
ATC*code	**Frequency**	**%**
C03 (Diuretics)	251	24.9
N06A (Antidepressants)	238	23.6
C01D (Vasodilators used in cardiac diseases)	136	13.5
C07 (Beta blocking agents)	129	12.8
C09 (Renin-angiotensin system inhibitors)	118	11.7
C08 (Calcium channel blockers)	64	6.3
N05A (Antipsychotics)	47	4.7
N04B (Dopaminergic agents)	22	2.2
G04CA (Alpha-adrenoreceptor antagonists)	4	0.4
C02 (Antihypertensives)	1	0.1
Total	1010	100

*Anatomical Therapeutic Chemical classification system.

## Discussion

### Main findings

Patients who had fallen were prescribed a higher number of continuous-use FRIDs than patients with no reported falls. A significant proportion (87%) of the study sample was taking FRIDs and ODs, as in other studies [27]. More men reported falls during the past three months; however, more women suffered from severe falls leading to emergency visits or hospital admission during the past year.

The study sample had a high incidence of both reported falls during the last three months (29%) and severe falls (17%). The results are similar to previously published results [28]. Data for reported falls during the past three months might have included severe falls and this explains the higher incidence of reported falls.

Fallers used a higher number of FRIDs, consistent with the findings of similar studies [29]. It is difficult to compare FRID data between different studies, since there are several different FRID classifications. Other international FRID lists include analgesics, hypoglycaemics and urinary antispasmodics [30-32]. Since drugs from these classes were not included in the Swedish NBHW lists, our results may differ from studies using more extensive FRID and OD lists. We chose not to merge the FRID and the OD lists from the NBHW but to present the results separately, because some drugs (e.g. antipsychotics) are classified both as FRIDs and as ODs.

Female sex and residency in nursing homes were associated with severe falls. Due to low bone mass, the presence of osteoporosis and low muscle strength, females are more likely than males to experience a fall-related injury [33,34]. In our study, female sex was associated with a higher number of FRIDs and this might explain the association with severe falls. Nursing home patients have increased care needs due to cognitive impairment, multiple illnesses and the use of a high number of drugs, and might therefore be more prone to fall. Numbers of FRIDs and ODs were associated with the total number of drugs and with severe falls. This is in agreement with previous studies showing strong evidence of an association between the use of psychoactive drugs and falls in the elderly [18], as well as between polypharmacy and falls [10,35,36].

A majority of the patients were females, lived in nursing homes and had a high number of drugs, as in other studies [37].

Antidepressants and anxiolytics were the most frequently used FRIDs and have been previously found to predispose elderly patients to falling [38]. The most frequently prescribed FRID in the study sample was oxazepam. Due to their muscle-relaxing effects, benzodiazepines have been associated with an increased risk of hip fractures in the elderly [39]. Cardiovascular drugs such as the commonly prescribed diuretic furosemide can cause or worsen orthostatic hypotension. However, there was no association between the numbers of ODs and falls in this study.

Almost a third of the patients complaining of moderate to severe symptoms of dizziness or unsteadiness reported falling in the three months prior to the study, compared to less than 10% of those who had no complaints. This suggests that the PHASE-20 symptom checklist might be a useful tool to predict falls among elderly patients. One strength of this study is that the drug lists are accurate and compliance with prescribed drug therapy was high due to the use of the MDD system. The reliability of the data is high since it was collected in a standardized manner by a single individual. The studied sample was from several different geographic regions in Skåne, Sweden, which increases the generalizability of our results. PHASE-20 was found to have acceptable consistency, test-retest reliability and internal validity [25]. Nurses that used the symptom checklist (PHASE-20) had direct contact with the patients, which ensured more accurate description of their symptoms.

### Limitations

A major limitation of the study is the cross-sectional design with collection of retrospective data about falls. Since no risk assessment tool was used, we are unable to stratify patients into low and high risk for falls. Data on patients’ diagnoses were not collected. It is therefore hard to draw a firm conclusion as to whether the cognitive impairment itself or the treatment of its symptoms is associated with falls.

Another major limitation of the study is also the lack of geriatric assessment. The identification of cognitive impairment, comorbidity and functional disability would clarify the contribution of other potential factors in increased fall risk.

Another limitation is that we assessed data about number of FRIDs and ODs regardless of the defined daily dosage of each drug. More detailed drug information might have provided better understanding of whether drug dosage affects fall risk.

All patients were included in the fall evaluation, even though some of them were not ambulatory. This may have caused some bias, since the chair-bound and bed-bound patients were not able to walk freely and were possibly less prone to falling.

Since the study is retrospective, it is not known what each study patient’s drug profile was during the period prior to the PHASE-20 evaluation.

### Future research

Interventions to optimize drug therapy in elderly patients with an emphasis on preventing falls would need to use a fall risk assessment tool including FRIDs to be able to stratify the patients into low and high risk of falling. A prospective study design would also confirm the strength of the association between exposure to FRIDs and subsequent falls.

All our patients used the MDD system. Although this system was originally developed to improve patient safety and drug compliance among those with multiple chronic co-morbidities, studies indicate that the use of the MDD system may be associated with a higher number of drugs, especially psychotropics [40], and poorer drug treatment. Future research should assess the possible effect of medication reviews with an emphasis on FRIDs and falls as a method to increase the quality of drug treatment in the elderly.

## Conclusions

Falls were common in this study sample. Nursing home patients and women had higher rates of falls requiring emergency room visits or hospitalisations. The number of FRIDs and ODs were associated with the total number of drugs. Fallers had a higher number of FRIDs but there was no association between number of ODs and falls. Antidepressants and anxiolytics were the most frequently used FRIDs. Interventions to prevent falls in elderly patients with a focus on reducing the total number of drugs and withdrawing psychotropic medications might improve the quality of drug treatment in elderly primary care patients.

## Abbreviations

ATC: Anatomical therapeutic chemical; FRIDs: Fall risk-increasing drugs; MDD: Multi-dose drug dispensing; NBHW: National board of health and welfare; ODs: Drugs that may cause or worsen orthostatism.

## Competing interests

The authors declare no competing interests.

## Authors’ contributions

VM, ÅB and MM participated in study design and coordination, data collection and statistical analysis, and drafted the manuscript. UJ, TW and PM participated in the design of the study and choosing of statistical analyses. All authors reviewed and approved the final manuscript.

## Pre-publication history

The pre-publication history for this paper can be accessed here:

http://www.biomedcentral.com/1471-2318/14/40/prepub

## Supplementary Material

Additional file 1 **PHASE-20. **PHArmacotherapeutical Symptom Evaluation, 20 questions. Click here for file
