# Supplementary material for: Fall risk-increasing drugs and falls: a cross-sectional study among elderly patients in primary care
Source: BMC Geriatr. 2014 Mar 27;14:40. doi: 10.1186/1471-2318-14-40 (PMC3986685; doi:10.1186/1471-2318-14-40)
Supplement: Additional file 1 — PHASE-20. PHArmacotherapeutical Symptom Evaluation, 20 questions. [file 1471-2318-14-40-S1.pdf]

PHASE-20: skattningsskala för möjliga läkemedelsrelaterade symtom

Bakgrundsdata

Namn patient	Personnummer

Boende	Kontaktuppgift sjuksköterska (namn, telefonnummer)

Uppgifter kring diagnos samt efterfrågade lab- och mätvärden noteras av ansvarig sjuksköterska.

Diagnoser

Under de tre senaste månaderna	Ja	Nej	Om ja, beskriv hur ofta det hänt samt trolig orsak
Konfusionsperiod			
Fallolycka			

		Datum			Datum
Längd			BT liggande		
Vikt			BT sittande/ stående		
S-kreatinin			Puls		

Är patienten	O Uppegående	O Rullstolsburen	O Sängbunden

Hur mycket har patienten kunnat delta i bedömningen?	O Helt	O Delvis	O Inte alls

Övriga kommentarer (exempelvis svårigheter att svälja tabletter)

PHASE-20: skattningsskala för möjliga läkemedelsrelaterade symtom

Datum	Kontaktuppgift vårdpersonal (namn, telefonnummer)

Sätt ett kryss i det alternativ som bäst motsvarar besvärsnivån under de senaste två veckorna.
Eventuella förtydligande lämnas i *kommentarer*, alternativ under *övriga kommentarer*.
Stryk under de symtom som stämmer bäst och ~~stryk över~~ de symtom som inte stämmer.

	Besvärsnivå (besvär)				
**Symtom**	**Inga**	**Små**	**Måttliga**	**Stora**	**Kommentarer:**
1. Yr/ostadig/faller lätt					
2. Trött/dåsig/orkeslös					
3. Sover dåligt/mardrömmar					
4. Ont i magen/ont i bröstet					
5. Huvudvärk					
6. Nedstämd					
7. Orolig/ångestfylld					
8. Lättirriterad					
9. Glömsk					
10. Dålig aptit					
11. Muntorr					
12. Illamående/kräks					
13. Diarré					
14. Förstoppning					
15. Hjärtklappning					
16. Svullna ben					
17. Andfådd					
18. Täta urinträngningar/ urinläckage					
19. Klåda/utslag					
20. Annat (till exempel smärta)					

PHASE-20 (PHArmacotherapeutical Symptom Evaluation, 20 frågor) är framtagen i samarbete mellan Läkemedelskommittén i Landstinget i Uppsala län och FoU Äldre, Regionförbundet Uppsala län. (*Hedström M, Lidström B, Hulter Åsberg K. (2009). PHASE-20: ett nytt instrument för skattning av möjliga läkemedelsrelaterade symtom hos äldre personer i äldreboende. Nordic Journal of Nursing Research and Clinical Studies (Vård i Norden); 4:9-14*).
Efterfrågad bakgrundinformation har justerats för Region Skånes räkning

Dokumentansvarig: Åsa Bondesson, Läkemedelsenheten, Koncernkontoret, Region Skåne Version 2011-03-28
